# Short-Term Complications After Laparoscopic Choledochal Cyst Radical Surgery: Prevention and Treatment

**DOI:** 10.3389/fsurg.2020.583210

**Published:** 2020-10-23

**Authors:** Jiachen Zheng, Zhihan Li, Yonqin Ye, Bin Wang

**Affiliations:** ^1^Department of Pediatric Surgery, Shantou University Medical College, Shantou, China; ^2^Department of General Surgery, Shenzhen Children's Hospital, Shenzhen, China

**Keywords:** choledochal cyst, laparoscopic radical surgery, prevention, short-term complications, treatment

## Abstract

**Background:** Shenzhen Children's Hospital is one of the first hospitals in mainland China to conduct the laparoscopic choledochal cyst radical surgery. We aimed to analyze the short-term complications of treating choledochal cyst with laparoscopic surgery and to provide recommendations to reduce complications.

**Methods:** A retrospective study was carried out from May 2010 to December 2017. The treatment process (preoperative preparation, surgical procedures, and treatment of the short-term complications), age at surgery, the length of surgery, and the length of stay were reviewed and analyzed.

**Results:** A total of 325 cases were included in this study. Four cases (1.2%) were converted to laparotomy. Twenty-three cases (7.1%) exhibited the short-term complications, including bile leakage occurred in nine cases (2.8%), chylous ascites in one case (0.3%), pancreatic fistula in two cases (0.6%), intestinal necrosis in one case (0.3%), hemorrhage in four cases (1.2%), internal hernia in two cases (0.6%), and stoma necrosis in four cases (1.2%). Among patients younger than 3 months old, two cases (10.5%, *P* < 0.05) were converted to laparotomy, and four cases (21.1%, *P* < 0.05) exhibited complications. These patients also had a longer operative time (204.9 ± 10.8 min, *P* < 0.05) and hospital stay (12.2 ± 0.7 d, *P* < 0.001).

**Conclusion:** In our study, the incidence of short-term complication after laparoscopic choledochal cyst radical surgery was relatively low. This procedure is a quite safe and effective for most patients, even for young children. However, patients younger than 3 months old may require extra attention during the treatment.

## Introduction

Choledochal cyst (CC) is a relatively rare bile duct malformation, and although multiple hypotheses have been suggested to explain its causes, the etiology is still vague. Cyst excision with Roux-en-Y hepaticojejunostomy is generally considered the optimal treatment approach (1–3). In recent years, due to the rapid development of laparoscopic techniques, laparoscopic surgery has been accepted as an effective and safe approach for the treatment of CC (4).

Shenzhen Children's Hospital is one of the first hospitals in mainland China to conduct the laparoscopic CC radical surgery. Until December 2017, 325 patients had undergone this surgery. We optimized our treatment process in terms of preoperative preparation, the surgical procedure, prevention and treatment of complications.

In this study, we reviewed every case of laparoscopic CC radical surgery performed in our hospital. We aimed to analyze the short-term complications of the treatment of the CC with laparoscopic surgery and to provide recommendations to reduce complications.

## Materials and Methods

### Patients

Data were collected from the case system of Shenzhen Children's Hospital. All cases were preoperatively diagnosed by ultrasound, computed tomography (CT) or magnetic resonance cholangiopancreatography (MRCP) according to the Todani classification (5). All included cases underwent laparoscopic radical surgery. For cases with severe infection, the surgery was postponed until the infection was managed. If fever or jaundice persisted with anti-infection therapy, then abdominal biliary drainage was performed first, and the surgery was performed next. The treatment process (including preoperative preparation, surgical procedures, and treatment of the short-term complications), age at surgery, the length of surgery, and the length of stay were recorded and analyzed. According to clinical observation, patients younger than 3 months who underwent this surgery showed a tendency to exhibit short-term complications; therefore, we divided the cohorts into two groups, patients younger than 3 months at surgery (cohort A) and those older than 3 months at surgery (cohort B). The study was reviewed and approved by the Ethics Committee of Shenzhen Children's Hospital.

### Statistical Analysis

SPSS 23.0 software was used for statistical processing. Differences between groups were compared with the *t*-test, Mann–Whitney U test and Wilcoxon signed rank test as appropriate. To determine statistical differences between ordinal values, the Chi-square or Fishers' exact test was used as appropriate. *P* < 0.05 was considered statistically significant.

### Preoperative Preparation

Anti-infection treatment, hypoalbuminemia correction, liver protection and vitamin K supplementation were applied. Patients with biliary infection and fever did not undergo laparoscopic surgery until their body temperature and leukocyte level returned to normal. If fever or jaundice persisted with anti-infection therapy, then abdominal biliary drainage was performed before surgery.

### Surgical Procedure

The patients were placed in the supine position under general anesthesia. A vertical incision through the umbilicus was performed to insert a 10 mm 30° laparoscope, and a carbon dioxide pneumoperitoneum was created (8–12 mmHg). Then, we placed trocars at the intersection of the right anterior axillary line and costal margin, the midpoint of the right rectus abdominis outer rim and the left upper abdominal rectus outer rim. The ligamentum teres hepatis was sutured and lifted externally to expose the porta hepatis. The ligamentum hepatoduodenal was cut with an electrotome, the gallbladder was dissected from its bed, and the cystic artery was ligated while temporarily maintaining the gallbladder. The duodenum was pushed down and then separated from the cyst antetheca. If the cyst was sufficiently small, it was dissociated completely using fine needle aspiration to reduce its size. The distal cyst wall was raised and closely separated at the confluence of the bile duct and pancreatic duct, then the distal bile duct was ligated, and then the distal cyst wall was resected in the pancreas. The paries posterior to the cyst to the junction of the porta hepatic and the common hepatic duct were dissected, and then the gallbladder was resected. After the incision, the common hepatic duct was opened to enlarge it, and if the left and right hepatic ducts were very narrow, these structures were also enlarged.

After the ligament of Treitz was identified, the jejunum was held with an intestinal grasper at 15 cm away from the Treitz ligament, the umbilical trocar was extracted, and the incision was expanded to 1.5–2.5 cm. The jejunum was removed from the body and excised, the distal jejunum was closed, and an additional 25–30 cm of the jejunum was removed, similar to a conventional laparotomy. After the end-to-side Roux-en-Y anastomosis of the proximal jejunum and distal jejunum side, these structures were placed back into the abdominal cavity. The mesenteric incision created for the Roux limb construction was closed. The trocar was repositioned, and the pneumoperitoneum was established. The jejunum was straightened out after anastomosis, and the Roux limb was moved up to the hepatic hilum behind the colon. After approximation of the jejunum and hepatic duct, a small incision was made on the antimesenteric side of the jejunum for end-to-side hepaticojejunostomy. The posterior and anterior rows of the hepaticojejunostomy were created using continuous sutures with the aid of 5–0 absorbable sutures. A drainage tube was placed near the anastomotic site, and finally the abdominal cavity was flushed and then closed.

## Results

### Patients

A total of 325 cases were included, cohort A included 19 cases, and cohort B included 306 cases. In cohort A, the age at surgery was 1.9 ± 0.7 months, while that in cohort B was 54.4 ± 19.6 months (*P* < 0.001) (Table 1).

**Table 1:** Surgical parameters in different cohorts.

	**Cohort A**	**Cohort B**	***P***
Age at surgery (m)	1.9 ± 0.7	54.4 ± 19.6	<0.001
Complications (*n*)	4 (21.1)	19 (6.2)	<0.05
Converted to laparotomy (*n*)	2 (10.5)	2 (0.7)	<0.05
Length of surgery (min)	204.9 ± 10.8	197.2 ± 11.7	<0.05
Length of stay (d)	12.2 ± 0.7	8.4 ± 0.6	<0.001

### Postoperative Complications: Incidence and Treatment

Among 325 cases, 321 children underwent laparoscopic CC excision and Roux-en-Y hepaticojejunostomy, and four cases (1.2%) were converted to laparotomy. One case suffered serious intestinal adhesion, which we considered to be caused by a previous radical intestinal atresia radical surgery. In one case, breathing was difficult to control with the pneumoperitoneum. The other two cases were converted to open surgery because of severe peritonitis.

Twenty-three cases (7.1%) exhibited short-term complications, including bile leakage occurred in nine cases (2.8%). Six cases recovered after abdominal drainage. Three cases underwent a second surgery for reanastomosis because of obstructed drainage (one by laparoscopic surgery and two by open surgery). One case (0.3%) suffered chylous fistula and recovered after abdominal drainage. Two cases (0.6%) suffered pancreatic fistula (by laparotomy). 2 cases (0.6%) had abdominal internal hernia at 6 days after surgery (by laparotomy). Necrosis of the partial intestine that acted as a replacement biliary duct occurred on one patient (laparotomy). Four cases (1.2%) suffered hemorrhage (two by laparoscopic surgery, two by open surgery). Four cases (1.2%) suffered stoma necrosis, all of whom received a second surgery due to the ileus. During the operation, we removed the necrotic segment and again performed Roux-en-Y anastomosis. No deaths occurred (Tables 2, 3).

**Table 2:** Short-term complications after laparoscopic choledochal cyst radical surgery.

	**Short-term complications**						
	**Bile leakage (*P* < 0.001)**	**Chylous ascites (*P* < 0.001)**	**Pancreatic fistula (*P* < 0.001)**	**Internal hernia (*P* < 0.001)**	**Intestinal necrosis (*P* < 0.001)**	**Hemorrhage (*P* < 0.001)**	**Stoma stenosis (*P* < 0.001)**
Cohort A (*n* = 19)	2 (10.5)	1 (5.3)	0	1 (5.3)	0	0	0
Cohort B (*n* = 306)	7 (2.3)	0	2 (0.7)	1 (0.3)	1 (0.3)	4 (1.3)	4 (1.3)
Total (*n* = 325)	9 (2.8)	1 (0.3)	2 (0.6)	2 (0.6)	1 (0.3)	4 (1.2)	4 (1.2)

**Table 3:** Treatment of the short-term complications.

**Short-term complications**	**Conservative treatment (*n*)**	**Second surgery (*****n*****)**	
		**Laparoscopy (*n*)**	**Laparotomy (*n*)**
Bile leakage	6	1	2
Chylous ascites	1	0	0
Pancreatic fistula	0	2	0
Internal hernia	0	0	2
Intestinal necrosis	0	0	1
Hemorrhage	0	2	2
Stoma stenosis	0	0	4
Totals	7 (30.5)	5 (21.7)	11 (47.8)

In cohort A, a total of four cases (21.1%) exhibited short-term complications. In cohort B, 19 cases (6.2%) exhibited short-term complications (*P* < 0.05). Two cases (10.5%) in cohort A and two cases (0.7%) in cohort B (*P* < 0.05) were converted to laparotomy (Table 1).

All patients with short-term complications such as pancreatic fistula, internal hernia, intestinal necrosis, hemorrhage, and stoma stenosis, all of them accepted a second surgery. For patients with bile leakage or chylous ascites, the conservative treatment was effective. In total, 69.5% of the patients required a second surgery, and 47.8% underwent laparotomy in the second surgery (Table 3).

### Length of Surgery and Length of Stay

The length of surgery was 204.9 ± 10.8 min in cohort A and 197.2 ± 11.7 min in cohort B (*P* < 0.05). The length of stay was 12.2 ± 0.7 days in cohort A and 8.4 ± 0.6 days in cohort B (*P* < 0.001) (Table 1).

## Discussion

In our center, most cases first showed the symptoms of pancreatitis or biliary calculi, which are common complications of CC and can usually be well managed by conservative treatment. When the symptoms cannot be well managed or the patient suffered biliary infection before, surgery should be performed immediately to prevent liver damage.

According to our experience, if the symptoms are alleviated after the conservative treatment, surgery is not urgent but is still a necessary step to resolve the recurrent pancreatitis and biliary calculi, etc. Usually, the patients undergo radical surgery a couple of weeks later to minimize the edema and adhesion caused by inflammation. However, the timing of surgery depends on many factors such as parental preferences. In some cases, parents would delay the surgery until the relevant symptoms returned.

In this study, we found that the patients in cohort A had a higher likelihood of short-term complications and conversion to laparotomy and a longer operative time and hospital stay. We found that the patients with a shorter history of CC had only mild inflammation and adhesion; therefore, we could easily and safely dissect the cyst. In the thin hepatic duct in infants, bile leakage can more easily occur in the anastomotic stoma. Excessively tight sutures cause stoma stenosis or necrosis, and appropriate traction tension should be noted during the surgery. In our study, the minimum diameter of the hepatic duct was 6 mm, which is quite large compared to the small abdominal cavity of the children. A limited operating space requires more advanced surgical skills, especially in laparoscopic surgery. In summary, patients younger than 3 months may require extra attention during the treatment.

Bile leakage is a severe short-term postoperative complication that mainly manifests as abdominal distention, fever and bile flow from the abdominal drainage tube 2–5 days after surgery. The likely explanations for this complication are as follows: (1) Drainage obstruction of the Y limb of the anastomotic stoma, which can cause dilation of biliary branches, and (2) deficiency of laparoscopic skills, improper suturing, pin-hole apart, and a high amount of anastomotic tension, etc. In our experience, performing another anastomosis instead of a repair can prevent recurrent bile leakage.

Chylous ascites is a rare postoperative complication caused by accidental lesions of the cisterna chyli or lymph trunk. This complication can be diagnosed by dyeing the liquid in the drainage tube white with Sudan III. One of our cases was a 2-month-old child diagnosed with a giant cyst. The cyst exceeded 10 cm in diameter and was located beyond the medioventral line with serious adhesion. The solution for chylous ascites is simple: postoperative fasting and intravenous nutrition.

In internal hernia, the Y limb enters the mesocolon gap because of peristalsis and usually results in the ileus. The possible causes are as follows: (1) Laparoscopic surgery influences peristalsis. (2) The ileocecal junction has not completely descended into the pelvic cavity completely. In addition, the enormous cyst extrudes from the ileocecal junction to the inferior liver margin, eventually causing the distal ileum to enter the mesocolon hole because the mesocolon hole is near the junction. (3) The mesocolon hole was not closed, the main reason for this complication. In our routine surgical process, to prevent intestinal limb anterograde peristalsis, we suture the bowel and mesentery; however, this step also causes the intestine to gather around the mesocolon below the hole. To improve blood supply, we do not suture the mesocolon hole, but the small intestine below the mesocolon hole can easily enter into the gap, leading to an internal hernia.

Intestinal necrosis is another rare and serious complication. The possible causes are as follows: (1) The intestine segment used to replace the bile duct was too long. To avoid reflux, we previously reserved 35 cm of the intestine, which also affects the absorption function of the small intestine and the blood supply. Then, we shortened the length of the intestine to 25 cm, and no reflux was observed. (2) The hole of the mesocolon is too small, causing ischemia and necrosis of the intestine. Due to the amplification effect under laparoscopy, the actual size of the hole is smaller than that observed. In subsequent operations, we enlarged the hole to guarantee sufficient blood supply.

Compared to data from other researches, in our study, patients had a lower likelihood of short-term complications, only 7.1% exhibited the short-term complications, and no deaths occurred. We also noticed that the types of complications were varied among the different medical centers. Remarkably, only one mortality case has been reported. In a Dutch study, one patient died within 30 days after the surgery due to an abdominal compartment syndrome combined with a congenital disorder of the glycosylation type Ia. For patients who underwent surgery in Fujian, China, the most common complication was biliary fistula, which is identical to our findings, but this result differed from that of patients who underwent surgery in Shandong, China. For the latter patients, the respiratory tract infection was the most common complication. In our study, no complications such as multiple intussusception, residual cyst in the distal end of the common bile duct, subcutaneous emphysema and wound infection were observed (6, 7).

Research from Shandong, China compared the operative time and blood loss between laparoscopy and laparotomy. In their study, although the operating time was longer for laparoscopy (170.3 ± 35.4 min for open surgery, 225.4 ± 51.0 min for laparotomy), but blood loss was much less in laparoscopy too. The similar results were also found in the In a Dutch research, the operating time for open surgery was 5.5 h for laparoscopy and 3.5 h for laparoscopy. Both researches indicated that there was no significant correlation between short-term complications and surgical approach.

The researches mentioned above also divided their patients by the age of 3 and 1 years, respectively, as we did, but they did not find any difference between the younger and older patients in the aspects of operative time and the length of the postoperative stay in the ward. But in a Dutch study, surgery before 1 year of age and laparoscopic surgery were associated with more postoperative long-term complications. However, in our study, we noted that patients under 3 months of age shown a higher possibility of short-term complications.

We believed that laparoscopic CC radical surgery is a quite safe and effective procedure for patients, even for young children. However, patients younger than 3 months may require additional attention during treatment.

## Data Availability Statement

The data analyzed in this study is subject to the following licenses/restrictions: This dataset is actually the case record system in our hospital which only accessible for staff inside. Requests to access these datasets should be directed to Bin Wang, szwb1967@126.com.

## Ethics Statement

Written informed consent was obtained from the minor(s)' legal guardian/next of kin for the publication of any potentially identifiable images or data included in this article.

## Author Contributions

JZ conducted a review of the literature and wrote and revised the original manuscript. ZL collected the data. YY reviewed the literature. BW reviewed and revised the manuscript prior to submission. All authors contributed to the article and approved the submitted version.

## Conflict of Interest

The authors declare that the research was conducted in the absence of any commercial or financial relationships that could be construed as a potential conflict of interest.
